# Supplementary material for: A self-supervised deep neural network for image completion resembles early visual cortex fMRI activity patterns for occluded scenes
Source: J Vis. 2021 Jul 14;21(7):5. doi: 10.1167/jov.21.7.5 (PMC8288063; doi:10.1167/jov.21.7.5)
Supplement: Supplement 1 [file jovi-21-7-5_s001.pdf]

A SELF-SUPERVISED DEEP NEURAL NETWORK FOR IMAGE COMPLETION RESEMBLES EARLY VISUAL CORTEX fMRI ACTIVITY PATTERNS FOR OCCLUDED SCENES
-
SUPPLEMENTARY MATERIAL

Michele Svanera*, **Andrew T. Morgan, Lucy S. Petro, Lars Muckli**
Centre for Cognitive Neuroimaging
Institute of Neuroscience and Psychology
University of Glasgow (UK)
`{name.surname}@glasgow.ac.uk`

May 18, 2021

VGG16 representation similarity

Similarity results between VGG16 layer activations and brain data are reported in Figure 1.

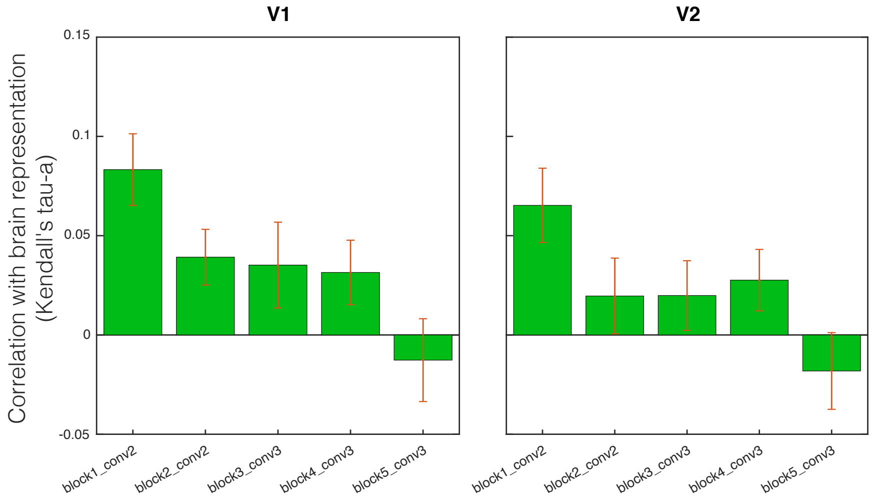

Figure 1: Comparison between brain and VGG16 RDMs. Averaged results across quadrants are shown.

In Fig. 1, results across areas are showing, accordingly with [Cichy et al., 2016, Güçlü and van Gerven, 2015], a decreasing of similarity going deeper in the network. The first layer, implementing mainly edge and color contrast detectors (low-level features), is overall the more similar with the brain. From the second, performance is different for every quadrant, which brings a correlation across the entire image to be low. Note that only the first three layers have receptive fields that are separable; from the fourth layer, it is not possible to disentangle quadrants anymore.

*Corresponding author: Michele.Svanera at glasgow.ac.uk

Encoder/decoder layers detail

Comparison between brain and aggregated encoder/decoder network RDMs are reported in Figure 2.

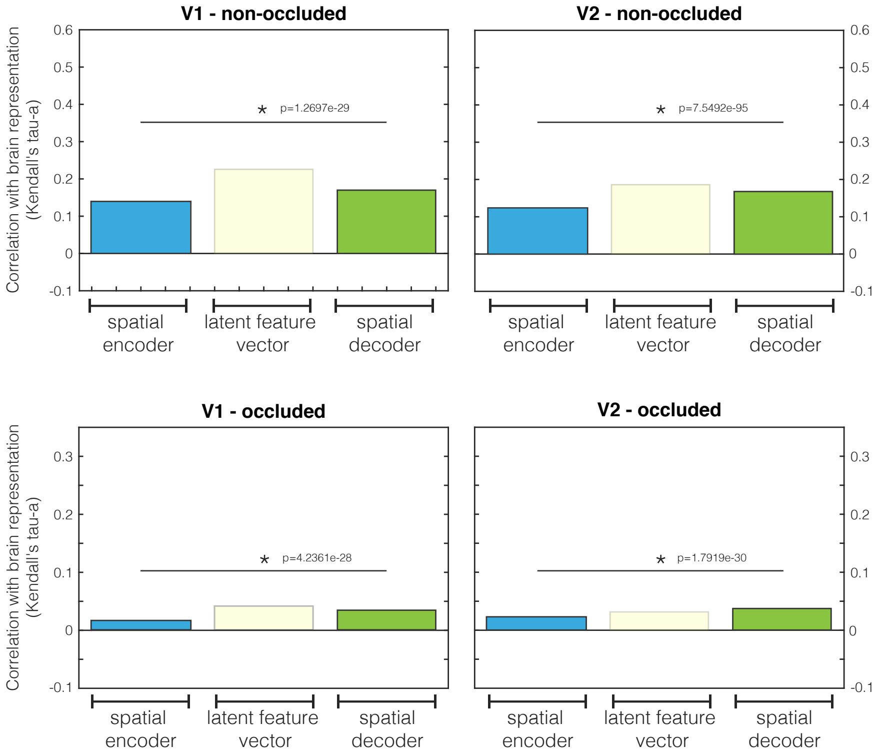

Figure 2: Comparison between brain and encoder/decoder network RDMs. First and second columns are different human visual areas, V1 and V2 respectively. First and second rows relate to non-occluded and occluded quadrants. Results are aggregate and displayed with different colours for spatial encoder, latent features, and spatial decoder network sections. Lines above bars show when the encoder and decoder populations difference is significant (Wilcoxon signed rank test). Latent feature vectors (yellow bars) are displayed in transparency for reference only: no comparison testing is performed on them.

Visual results on MNIST dataset

For sake of completeness, we trained and tested a model on MNIST dataset with occlusions. The steps performed are the same as in the manuscript: we downloaded the dataset, we applied the occlusion, we trained the model, and we tested it on a different set (also in this example we split training/validation/testing sets). Results in Figure 3 show how the method can learn well the data distribution, as long as it is trained and tested on the same population. Zooming in, it is possible to spot small differences, even if network outputs are very similar to the original image and indistinguishable. The good results derive from the task being relatively simple and from the high capability of the model.

Encoder/decoder activation functions

Encoder layers implement rectified linear unit (ReLu) function $y = f(x) = 0.5 * x + 0.5 * |x|$ and decoder leaky ReLu $y = f(x) = 0.6 * x + 0.4 * |x|$. In Figure 4 are shown the two activations.

Training and testing approach

The followed procedure to train our model, takes advantages of two networks: a `generator` (our encoder/decoder in Figure 2), which takes an occluded image as input and produces a reconstructed image in output, and a `discriminator`, which has to detect if the synthetised image is real or fake. In testing, only the generator is used, which, starting from

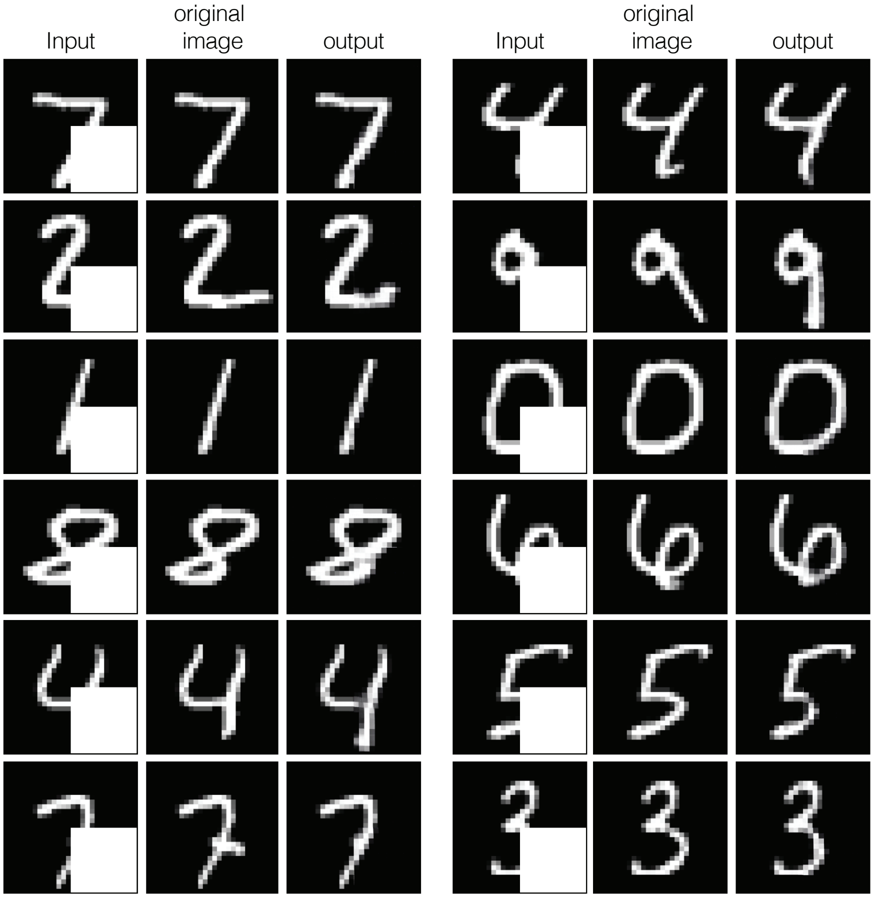

Figure 3: Visual results obtained on MNIST dataset. Images used in this figure were not part of the training set.

occluded images never seen before (not in the training set), synthesises their fully reconstructed versions. Outlines of training and testing procedures are shown in the Figure 5.

Increase and decrease of similarity in spatial encoders and decoders

We here performed a statistical analysis to support the claim on page 12: "progressive encoding layers become increasingly better descriptions of brain activity patterns". To test the significance of an increase (or decrease), we fit a linear regression model and display the p-value found with the t-statistic on the hypothesis test that the corresponding coefficient (the slope) is equal to zero or not. Figure 6 shows the results of this analysis; we displayed p-Values obtained in top right corners, coloring them in green or red for passing or failing the t-test, respectively ($p < 0.05$). As an example, the p-value of the t-statistic for the top left subplot (V1, non-occluded, spatial encoder) is $p = 1.6449e - 13$, which is smaller than 0.05, so this term is significant at the 5% significance level. As it is possible to notice, in every condition (V1 and V2, for occluded and non-occluded, 4/4) we have a statistically significant increase of similarity for spatial encoders. For spatial decoder, we have significant decreases for three cases over four (V2 occluded is not significant). For latent feature vectors, we do not have any significant results, which means that the slopes found are not statistically different than zero.

Experiment images

The 24 images used in the experiment are reported in Figure 7.

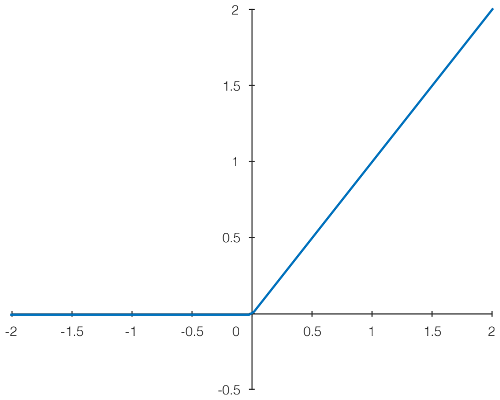

(a) ReLu - Encoder

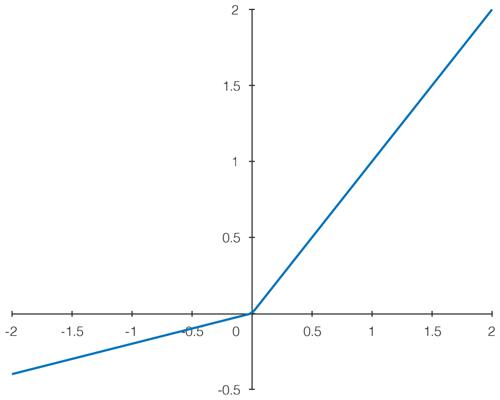

(b) Leaky ReLu - Decoder

Figure 4: Activation functions for (a) encoder and (b) decoder.

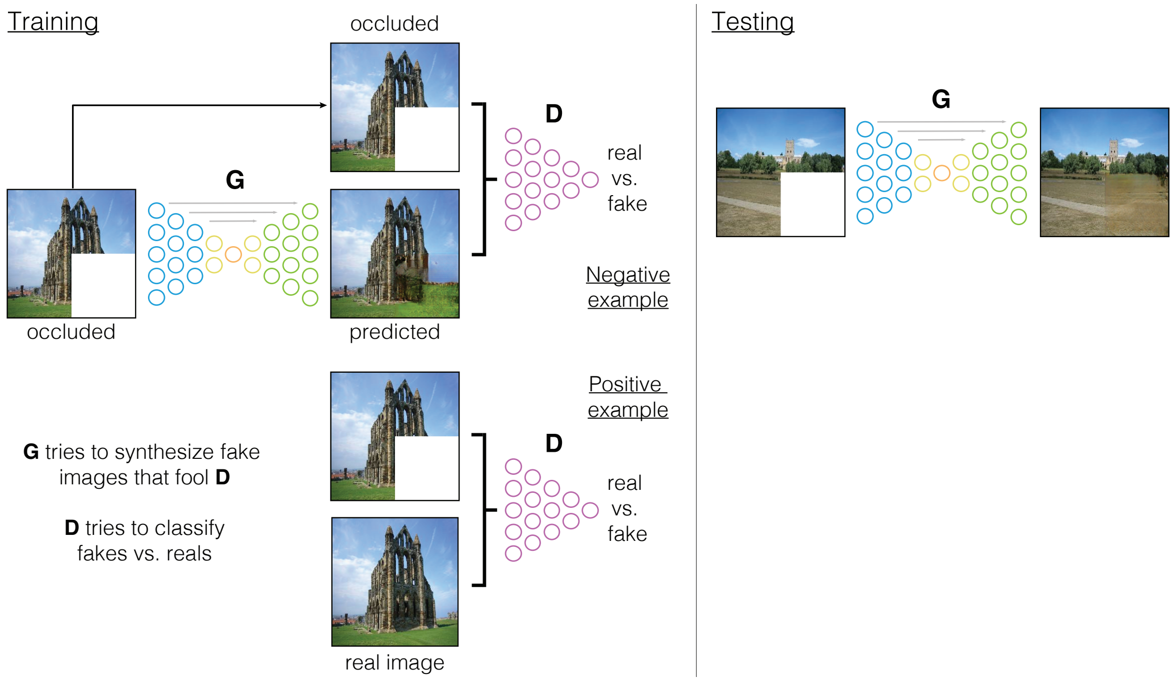

Figure 5: Training and testing procedures of the model. Deliberately inspired by Fig.2 of [Isola et al., 2017].

Model summaries

VGG16

In Table 1, VGG16 network details, with activation and receptive field dimensions, are presented. In blue the analysed layers.

Encoder/decoder

In Table 2, encoder/decoder network details, with activation and receptive field dimensions, are presented.

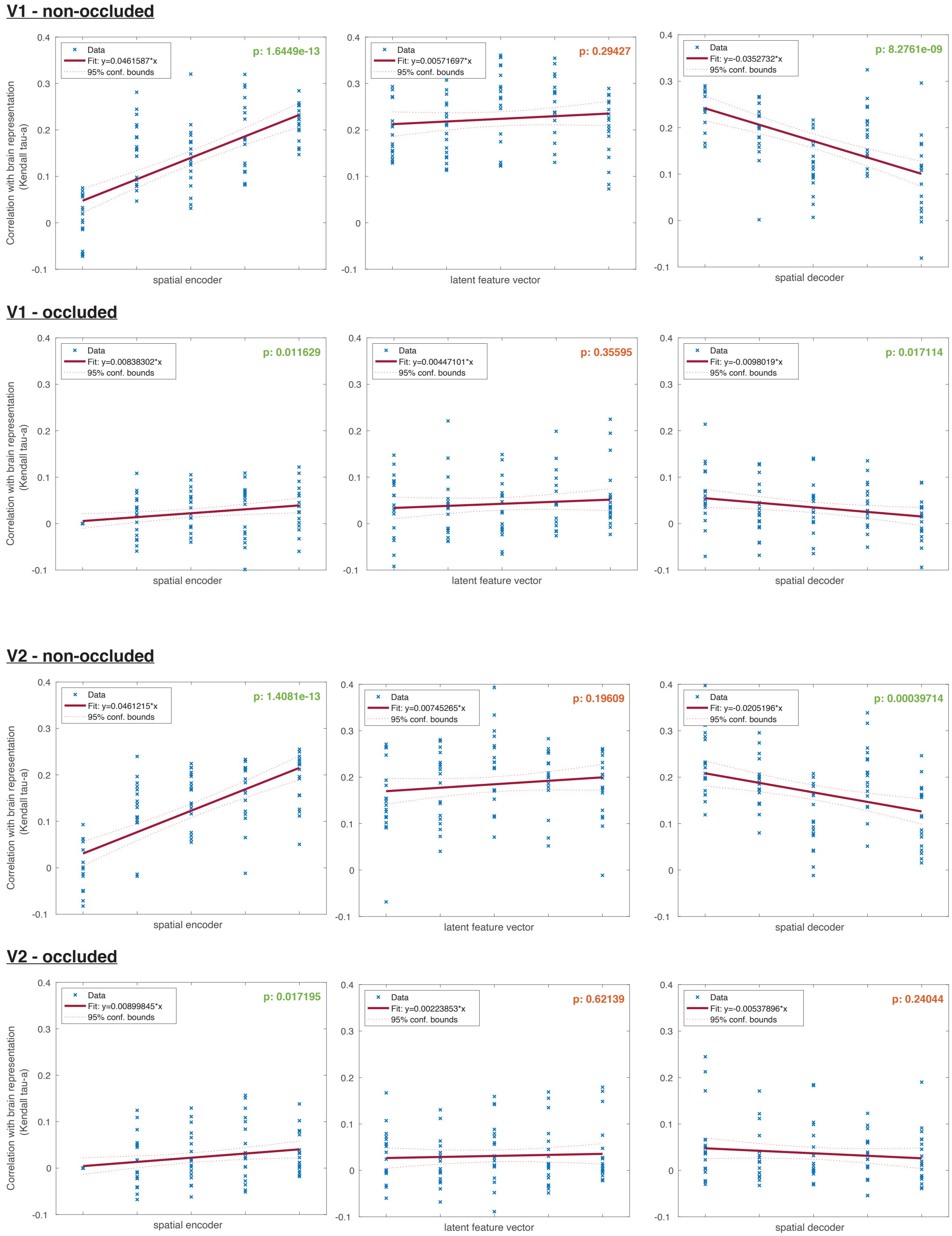

Figure 6: Statistical analysis to support the sentence: "progressive encoding layers become increasingly better descriptions of brain activity patterns". Every row displays results for a specific visual area and quadrant tested: V1 non-occluded, V1 occluded, V2 non-occluded, and V2 occluded. Each column shows results for spatial encoders, latent feature vectors, and spatial decoders respectively. To test the significance of an increase (or decrease), we fit a linear regression model (red line) using correlation data (every ?x? is a subject) and display the p-value found with the t-statistic on the hypothesis test that the corresponding coefficient (the slope) is equal to zero or not. The p-values are colored in green or red for passing or failing the t-test, respectively ($p < 0.05$).

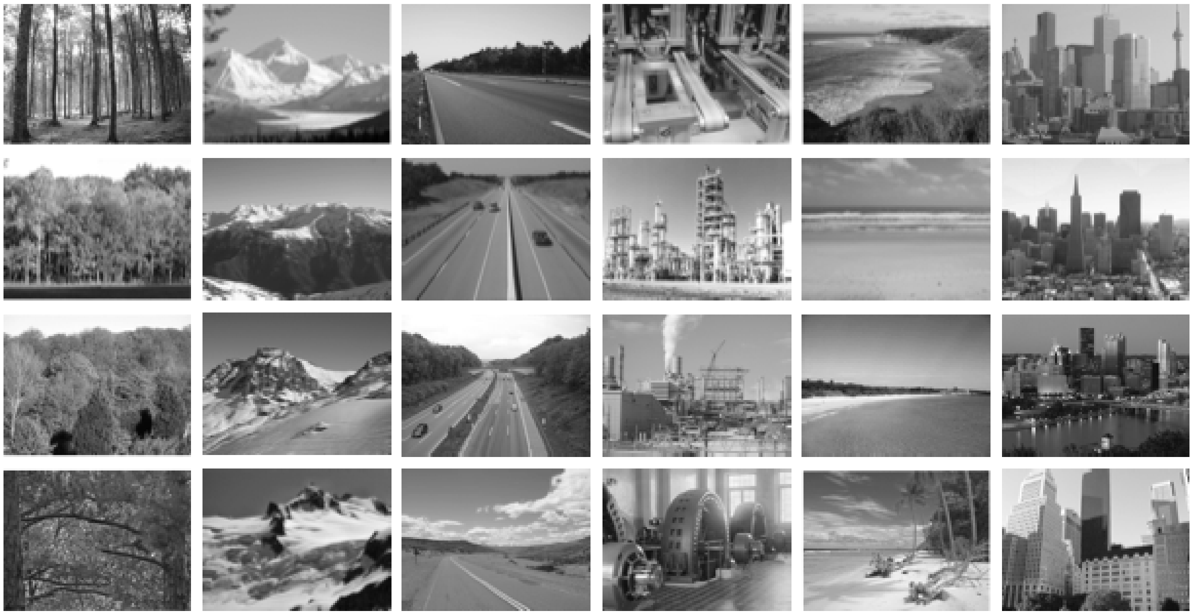

Figure 7: The 24 images (of 6 categories: forests, mountains, highways, industry, beaches, and buildings) used in the experiment. Images are taken from SUN database [Xiao et al., 2010].

Layer visualisation

Layer visualisation encoder/decoder

In Figures 8 to 15 are shown layer activations for `encoder_1` to `encoder_8`. Every Figure corresponds to a specific layer and it has two subplots. **(A)** Once a specific channel of the layer analysed is selected (randomly, the number is reported at the top), the top five activations are shown in column. **(B)** depicts the images where these patches are taken from, with red bounding boxes that indicate the location of the patches in the image.

Layer visualisation VGG16

In Figures 16 to 20 are shown layer activations for `block1_conv2`, `block2_conv2`, `block3_conv3`, `block4_conv3`, and `block5_conv3`. Every Figure corresponds to a specific layer and it has two subplots. **(A)** Once a specific channel of the layer analysed is selected (randomly, the number is reported at the top), the top five activations are shown in column. **(B)** depicts the images where these patches are taken from, with red bounding boxes that indicate the location of the patches in the image.

References

[Cichy et al., 2016] Cichy, R. M., Khosla, A., Pantazis, D., Torralba, A., and Oliva, A. (2016). Comparison of deep neural networks to spatio-temporal cortical dynamics of human visual object recognition reveals hierarchical correspondence. *Scientific reports*, 6.

[Güçlü and van Gerven, 2015] Güçlü, U. and van Gerven, M. A. J. (2015). Deep neural networks reveal a gradient in the complexity of neural representations across the ventral stream. *The Journal of Neuroscience*, 35(27):10005–10014.

[Isola et al., 2017] Isola, P., Zhu, J.-Y., Zhou, T., and Efros, A. A. (2017). Image-to-image translation with conditional adversarial networks. *CVPR*.

[Xiao et al., 2010] Xiao, J., Hays, J., Ehinger, K. A., Oliva, A., and Torralba, A. (2010). Sun database: Large-scale scene recognition from abbey to zoo. In *Computer vision and pattern recognition (CVPR), 2010 IEEE conference on*, pages 3485–3492. IEEE.

Table 1: VGG16 activation dimensions. Total params: $\sim$ 138M

Layer (type)	Output Shape	Receptive field
input image	(224, 224, 3)	n.a.
block1_conv1 (Conv2D)	(224, 224, 64)	(3, 3)
block1_conv2 (Conv2D)	(224, 224, 64)	(5, 5)
block1_pool (MaxPooling2D)	(112, 112, 64)	(6, 6)
block2_conv1 (Conv2D)	(112, 112, 128)	(10, 10)
block2_conv2 (Conv2D)	(112, 112, 128)	(14, 14)
block2_pool (MaxPooling2D)	(56, 56, 128)	(16, 16)
block3_conv1 (Conv2D)	(56, 56, 256)	(24, 24)
block3_conv2 (Conv2D)	(56, 56, 256)	(32, 32)
block3_conv3 (Conv2D)	(56, 56, 256)	(40, 40)
block3_pool (MaxPooling2D)	(28, 28, 256)	(44, 44)
block4_conv1 (Conv2D)	(28, 28, 512)	(60, 60)
block4_conv2 (Conv2D)	(28, 28, 512)	(76, 76)
block4_conv3 (Conv2D)	(28, 28, 512)	(92, 92)
block4_pool (MaxPooling2D)	(14, 14, 512)	(100, 100)
block5_conv1 (Conv2D)	(14, 14, 512)	(132, 132)
block5_conv2 (Conv2D)	(14, 14, 512)	(164, 164)
block5_conv3 (Conv2D)	(14, 14, 512)	(196, 196)
block5_pool (MaxPooling2D)	(7, 7, 512)	(212, 212)
flatten (Flatten)	(25088)	n.a.
fc1 (Dense)	(4096)	n.a.
fc2 (Dense)	(4096)	n.a.
predictions (Dense)	(1000)	n.a.

Table 2: Encoder/decoder activation dimensions. Total params: $\sim$ 228M

**Layer (type)**	**Output Shape**	**Receptive field**
input image	(256, 256, 3)	n.a.
encoder_1	(128, 128, 128)	(4, 4)
encoder_2	(64, 64, 256)	(10, 10)
encoder_3	(32, 32, 512)	(22, 22)
encoder_4	(16, 16, 1024)	(46, 46)
encoder_5	(8, 8, 1024)	(94, 94)
encoder_6	(4, 4, 1024)	(190, 190)
encoder_7	(2, 2, 1024)	n.a.
encoder_8	(1, 1, 1024)	n.a.
decoder_8	(2, 2, 1024)	n.a.
decoder_7	(4, 4, 1024)	n.a.
decoder_6	(8, 8, 1024)	n.a.
decoder_5	(16, 16, 1024)	n.a.
decoder_4	(32, 32, 512)	n.a.
decoder_3	(64, 64, 256)	n.a.
decoder_2	(128, 128, 128)	n.a.
output image	(256, 256, 3)	n.a.

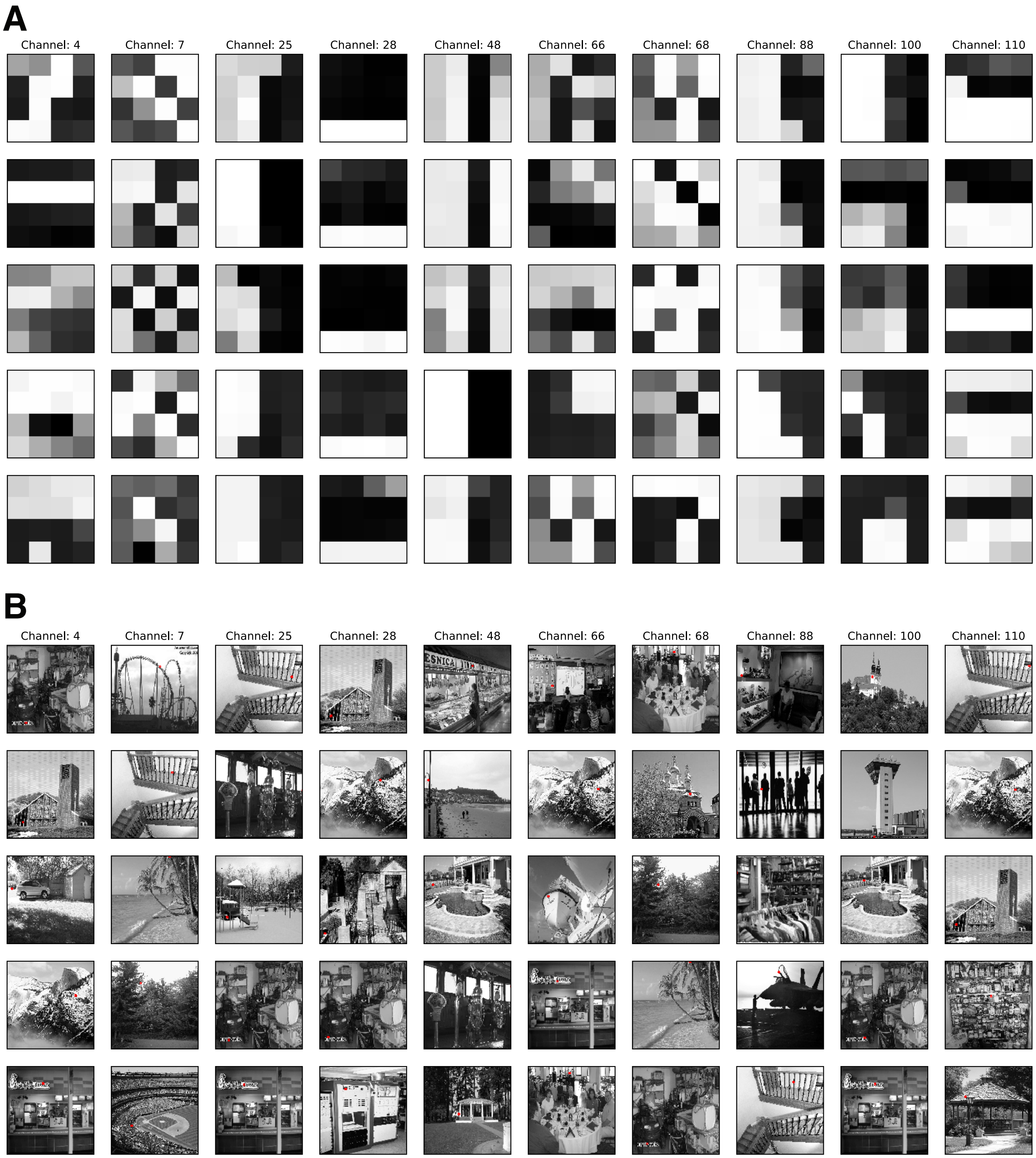

Figure 8: Layer `encoder_1` max activations. Every column is a channel. **(A)** Receptive fields, in the image space, that provide the five largest activations for a specific channel. **(B)** Original images are provided.

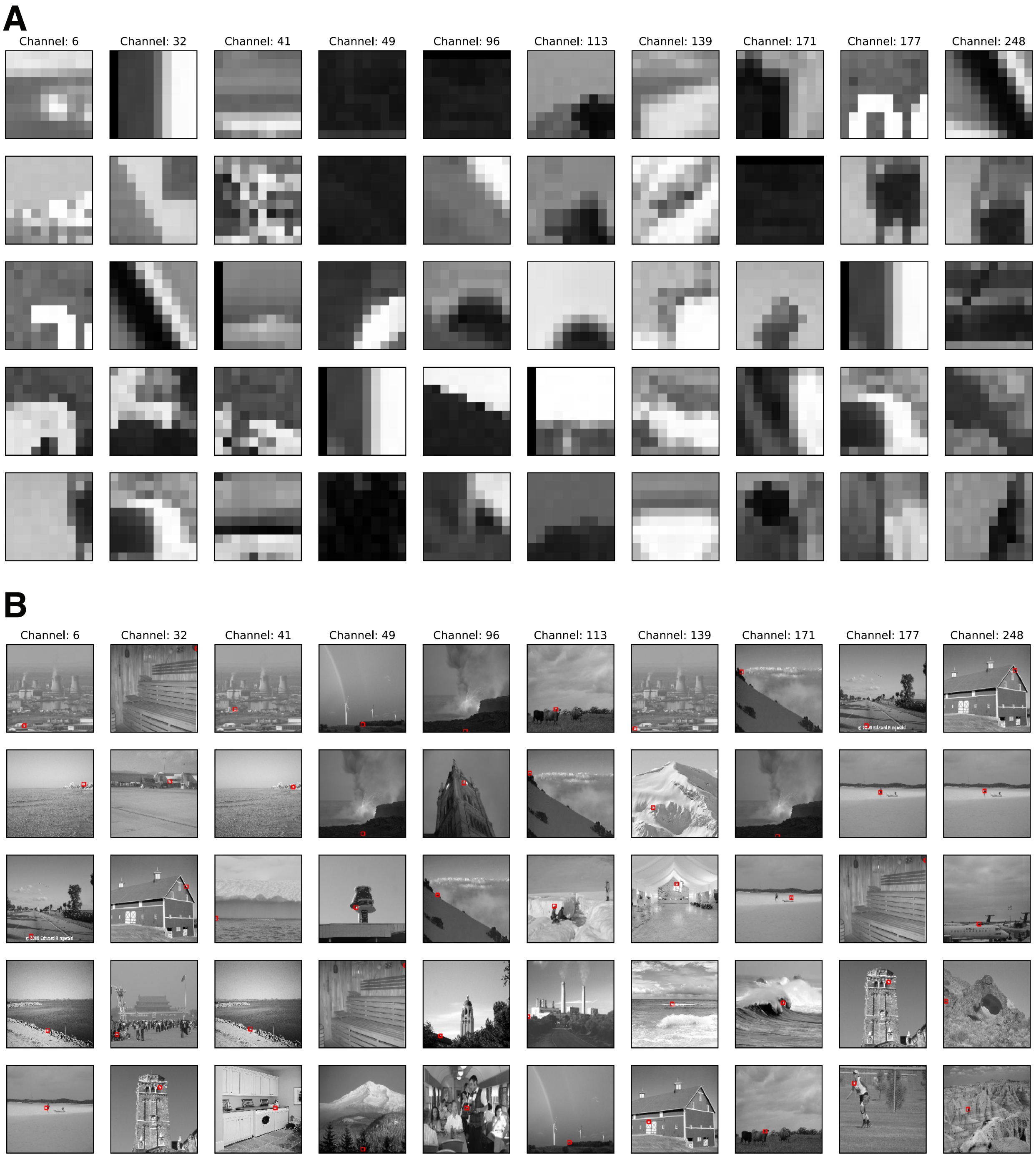

Figure 9: Layer `encoder_2` max activations. Every column is a channel. **(A)** Receptive fields, in the image space, that provide the five largest activations for a specific channel. **(B)** Original images are provided.

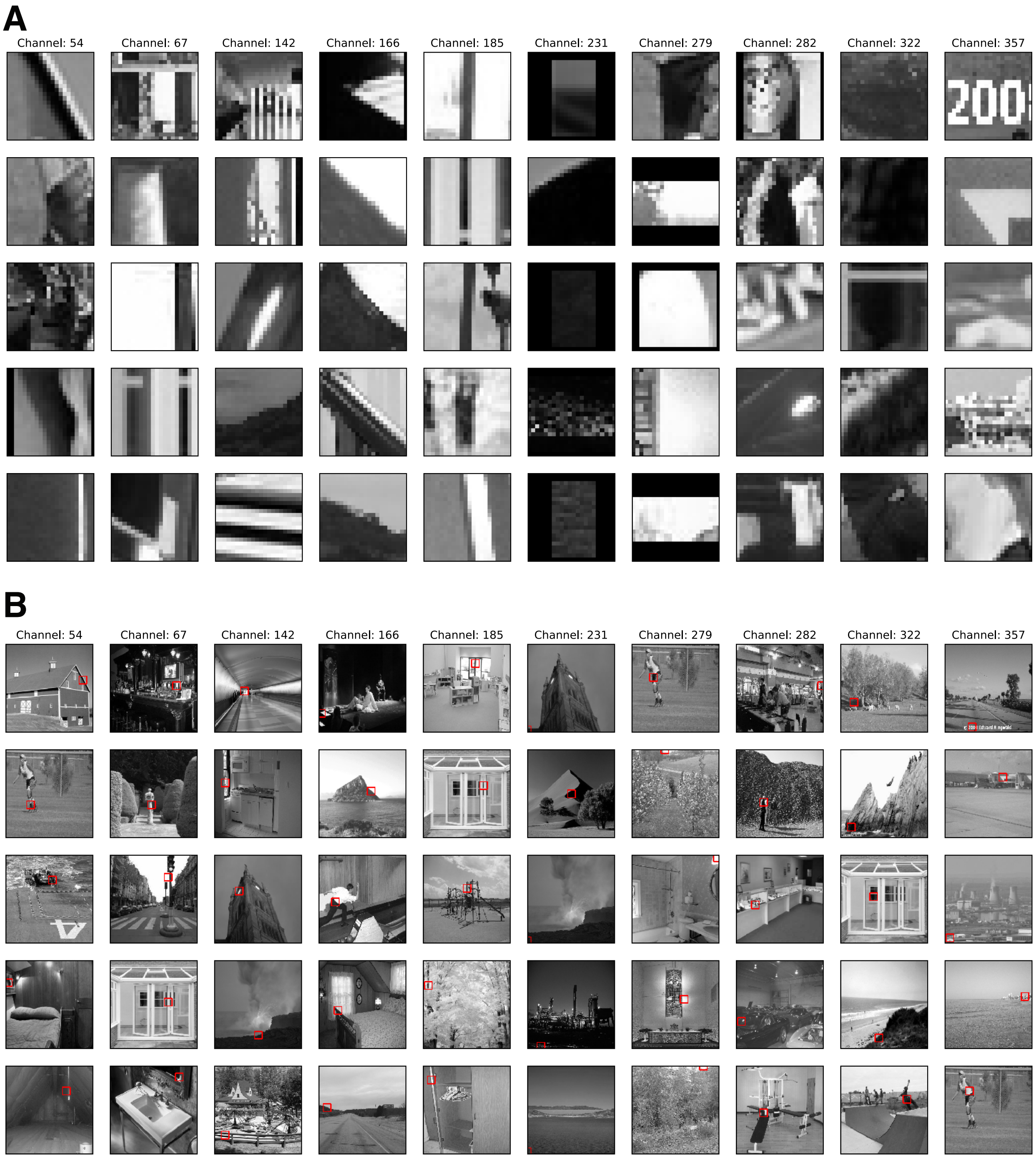

Figure 10: Layer `encoder_3` max activations. Every column is a channel. **(A)** Receptive fields, in the image space, that provide the five largest activations for a specific channel. **(B)** Original images are provided.

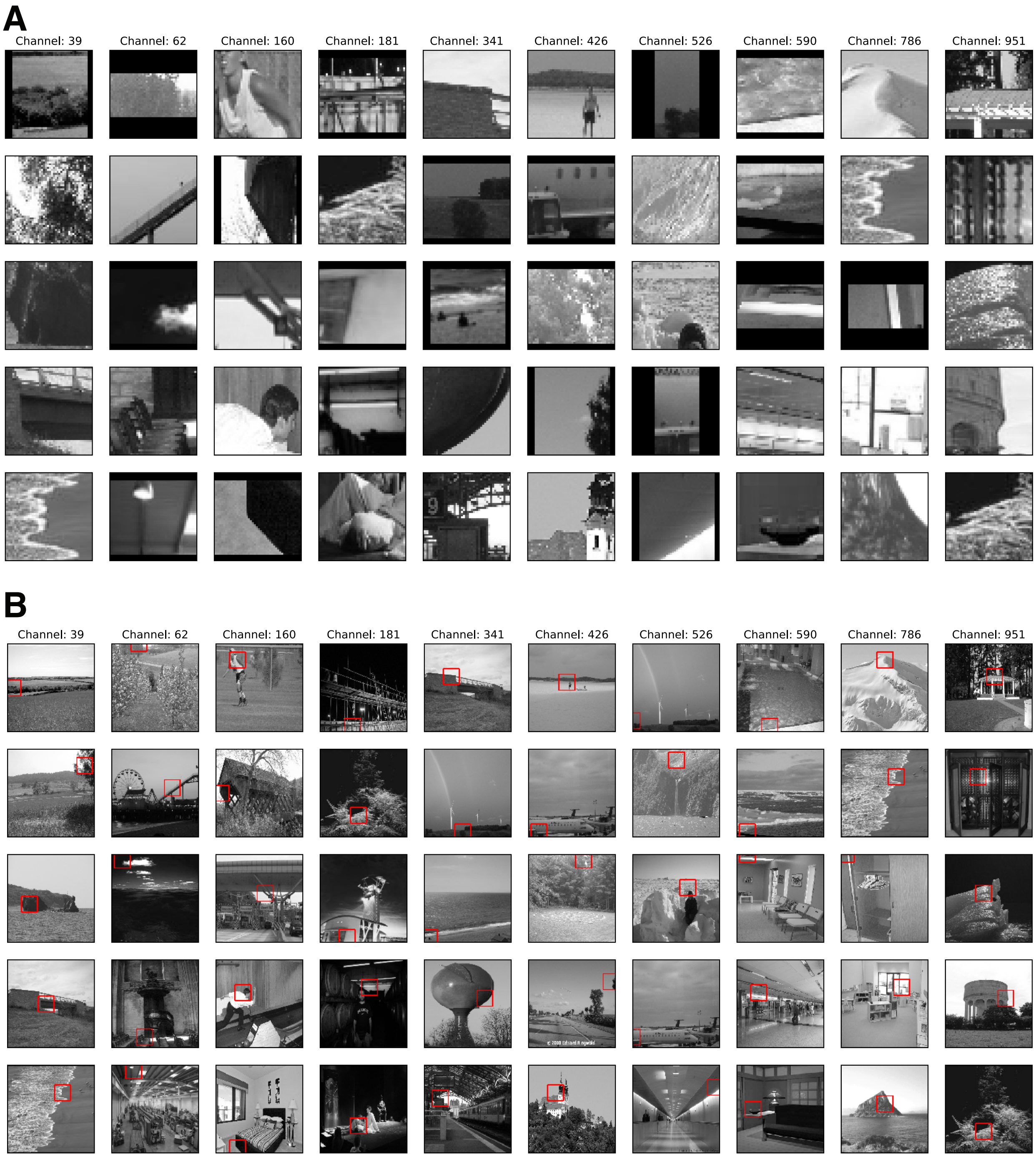

Figure 11: Layer `encoder_4` max activations. Every column is a channel. **(A)** Receptive fields, in the image space, that provide the five largest activations for a specific channel. **(B)** Original images are provided.

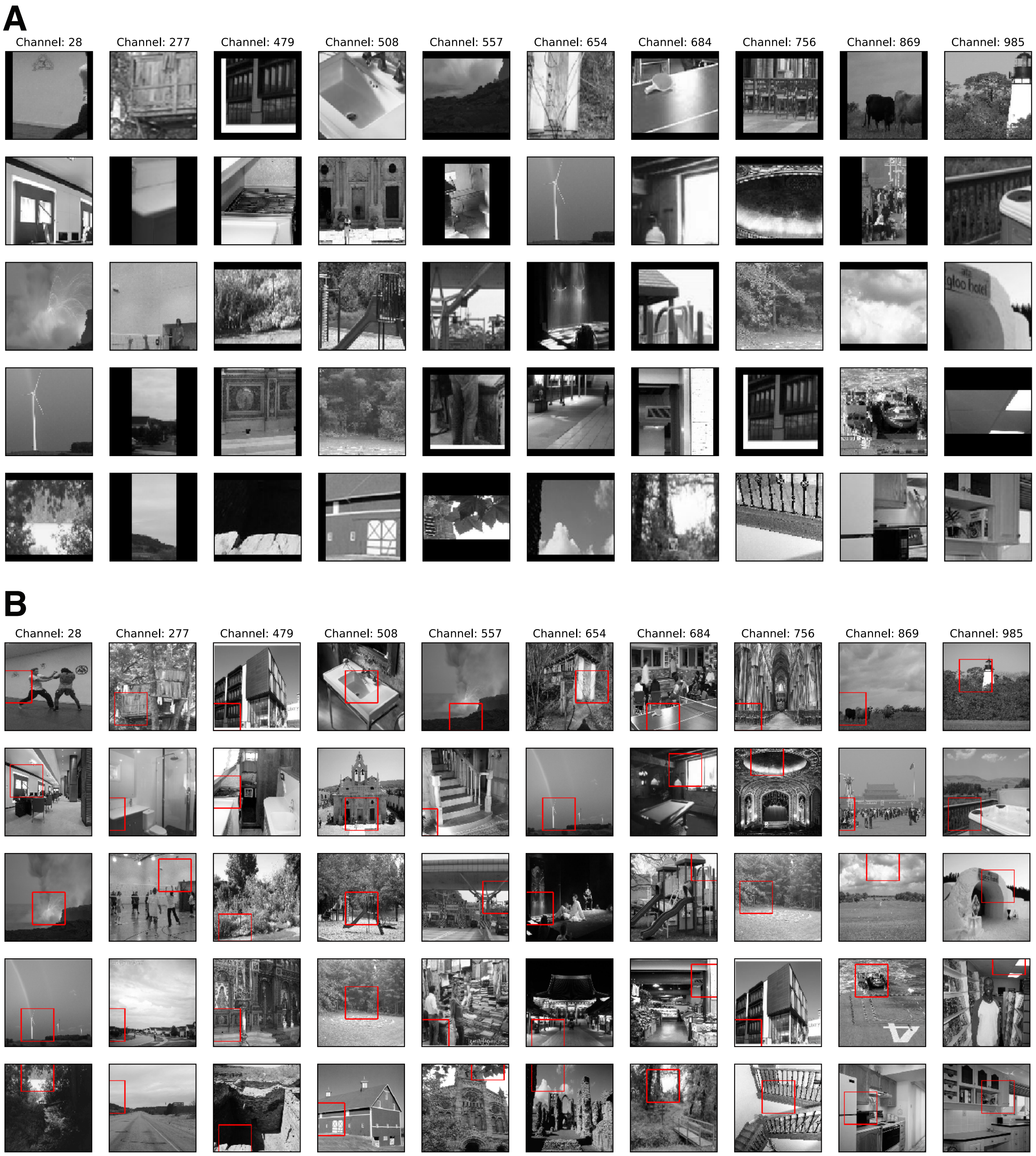

Figure 12: Layer `encoder_5` max activations. Every column is a channel. **(A)** Receptive fields, in the image space, that provide the five largest activations for a specific channel. **(B)** Original images are provided.

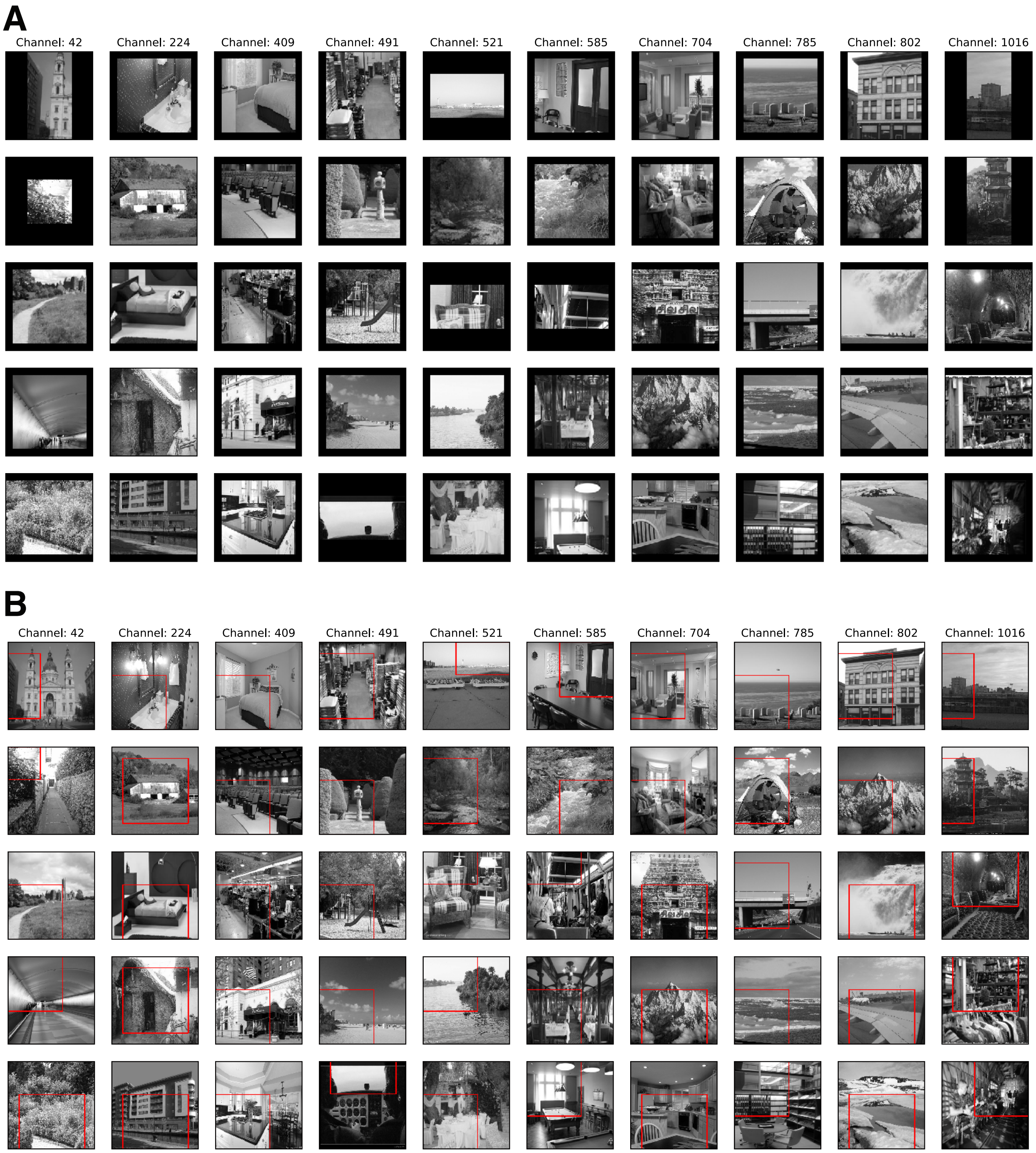

Figure 13: Layer `encoder_6` max activations. Every column is a channel. **(A)** Receptive fields, in the image space, that provide the five largest activations for a specific channel. **(B)** Original images are provided.

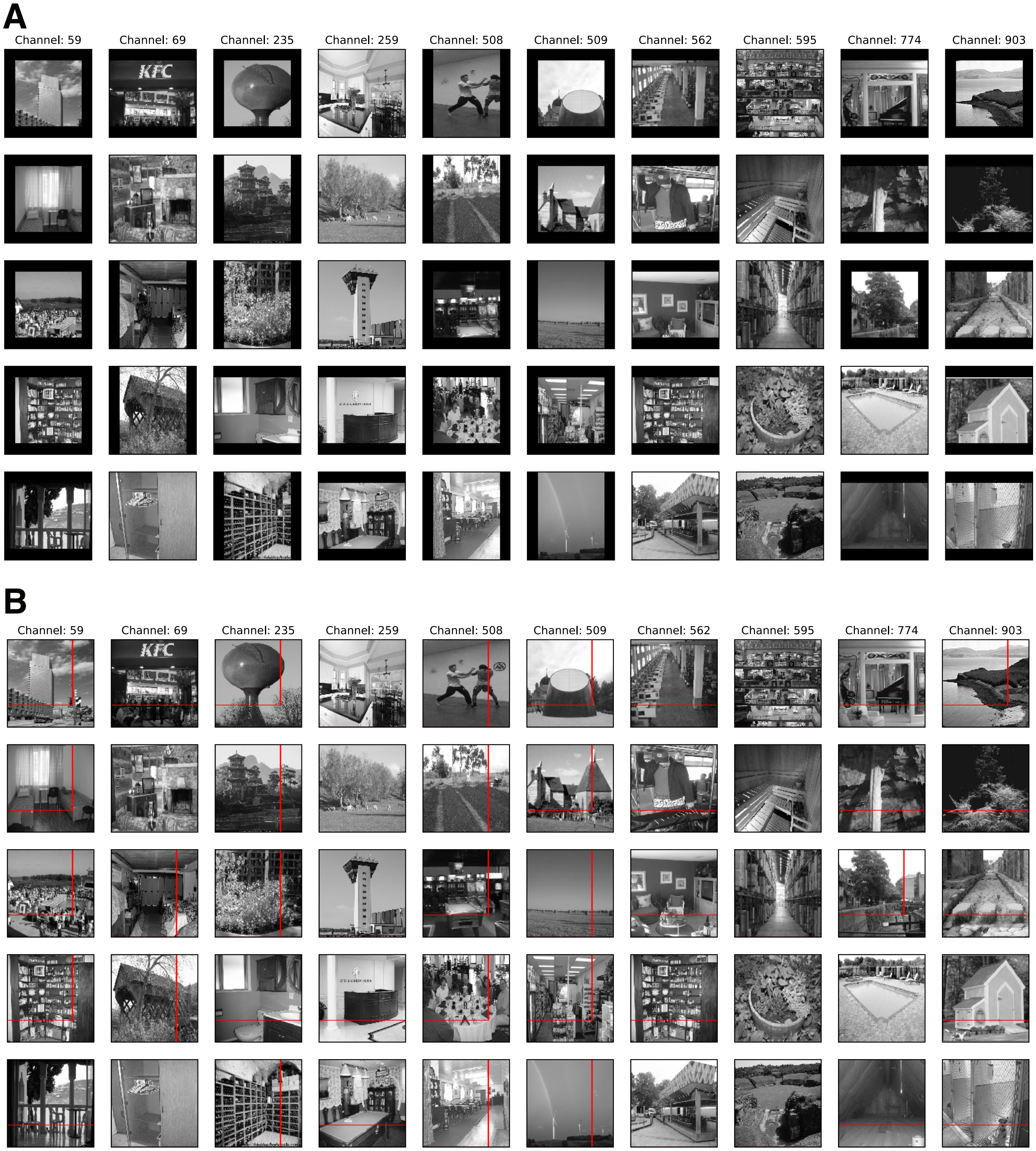

Figure 14: Layer `encoder_7` max activations. Every column is a channel. **(A)** Receptive fields, in the image space, that provide the five largest activations for a specific channel. **(B)** Original images are provided.

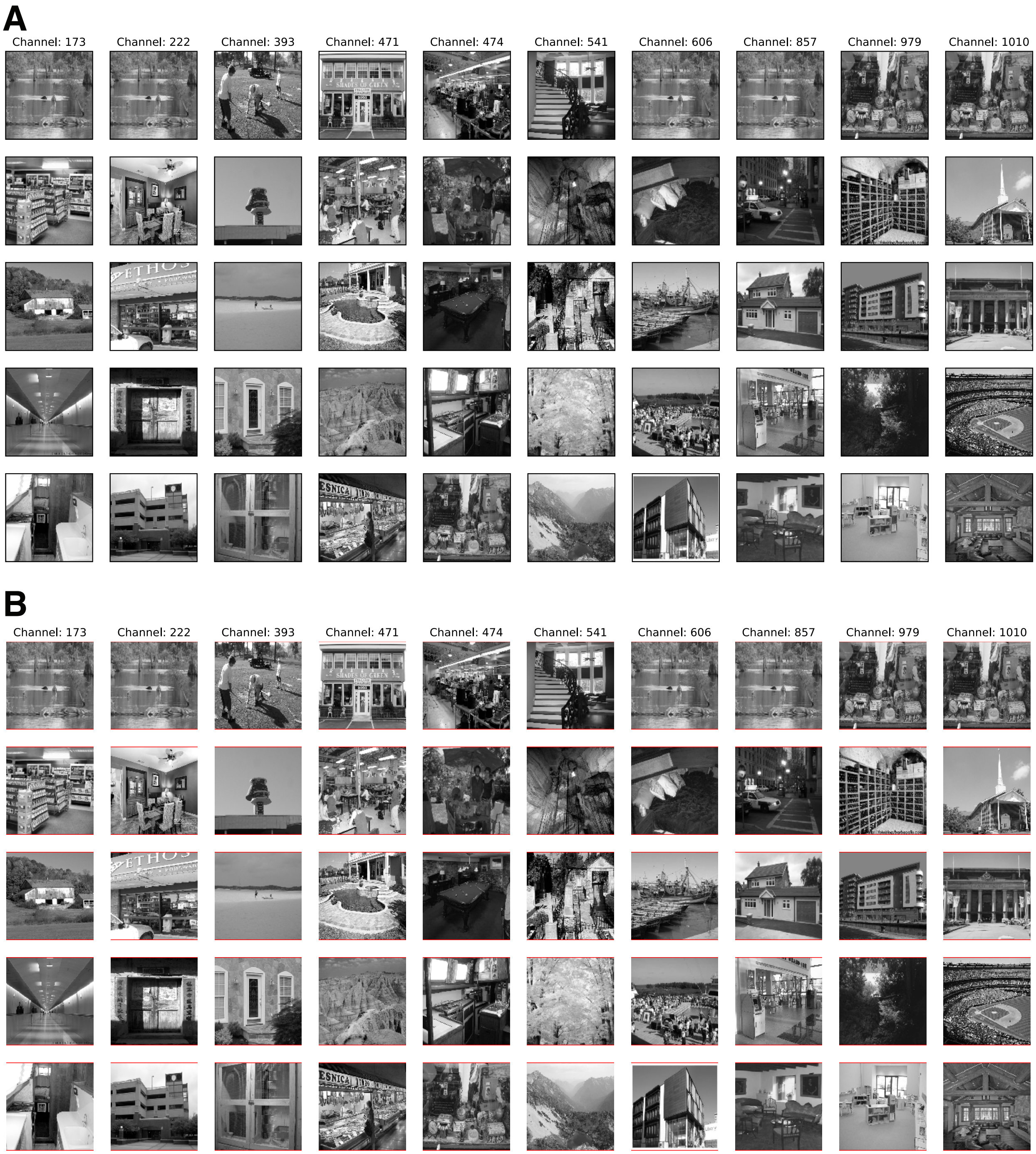

Figure 15: Layer `encoder_8` max activations. Every column is a channel. **(A)** Receptive fields, in the image space, that provide the five largest activations for a specific channel. **(B)** Original images are provided.

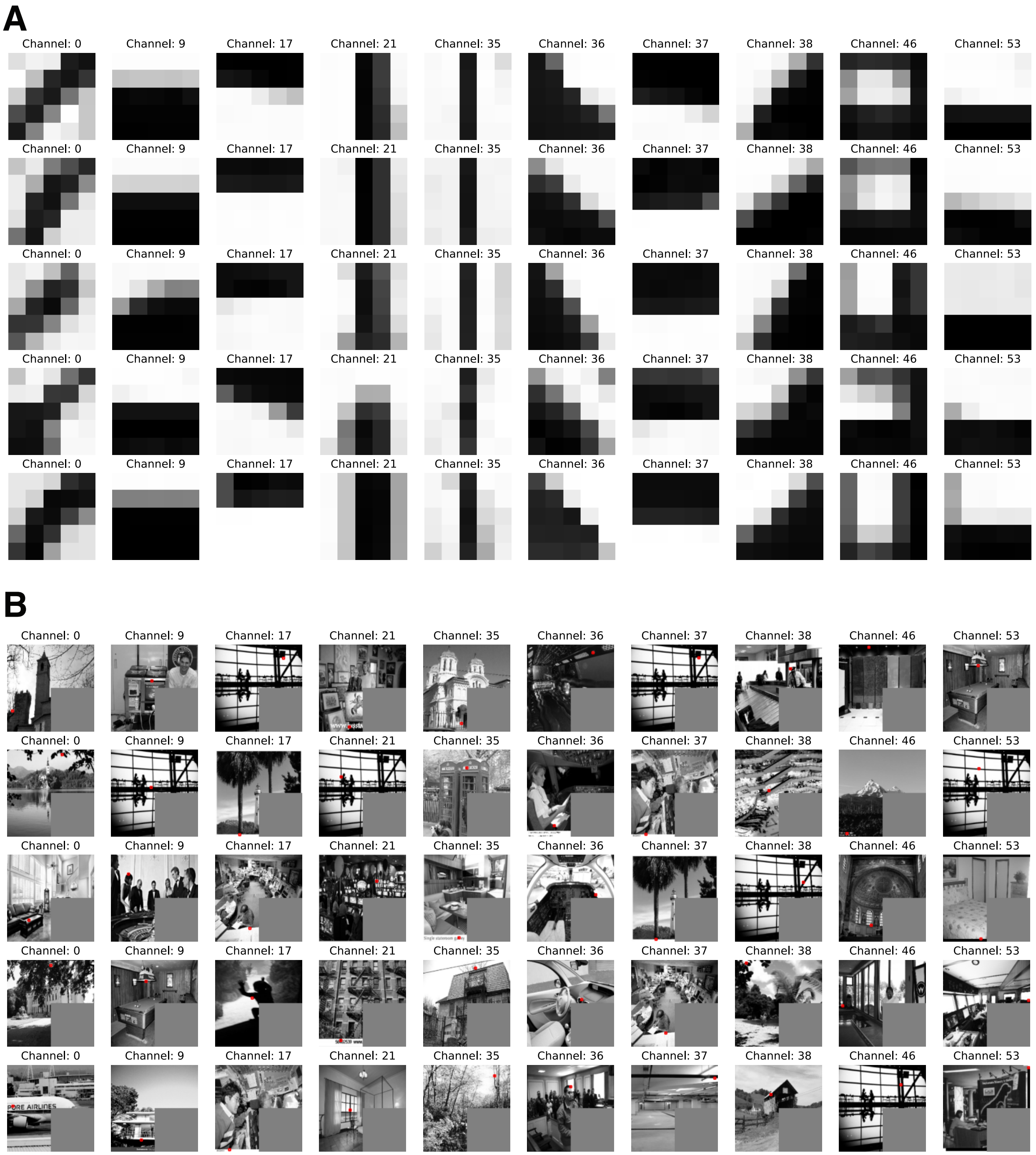

Figure 16: Layer `block1_conv2` max activations. Every column is a channel. **(A)** Receptive fields, in the image space, that provide the five largest activations for a specific channel. **(B)** Original images are provided.

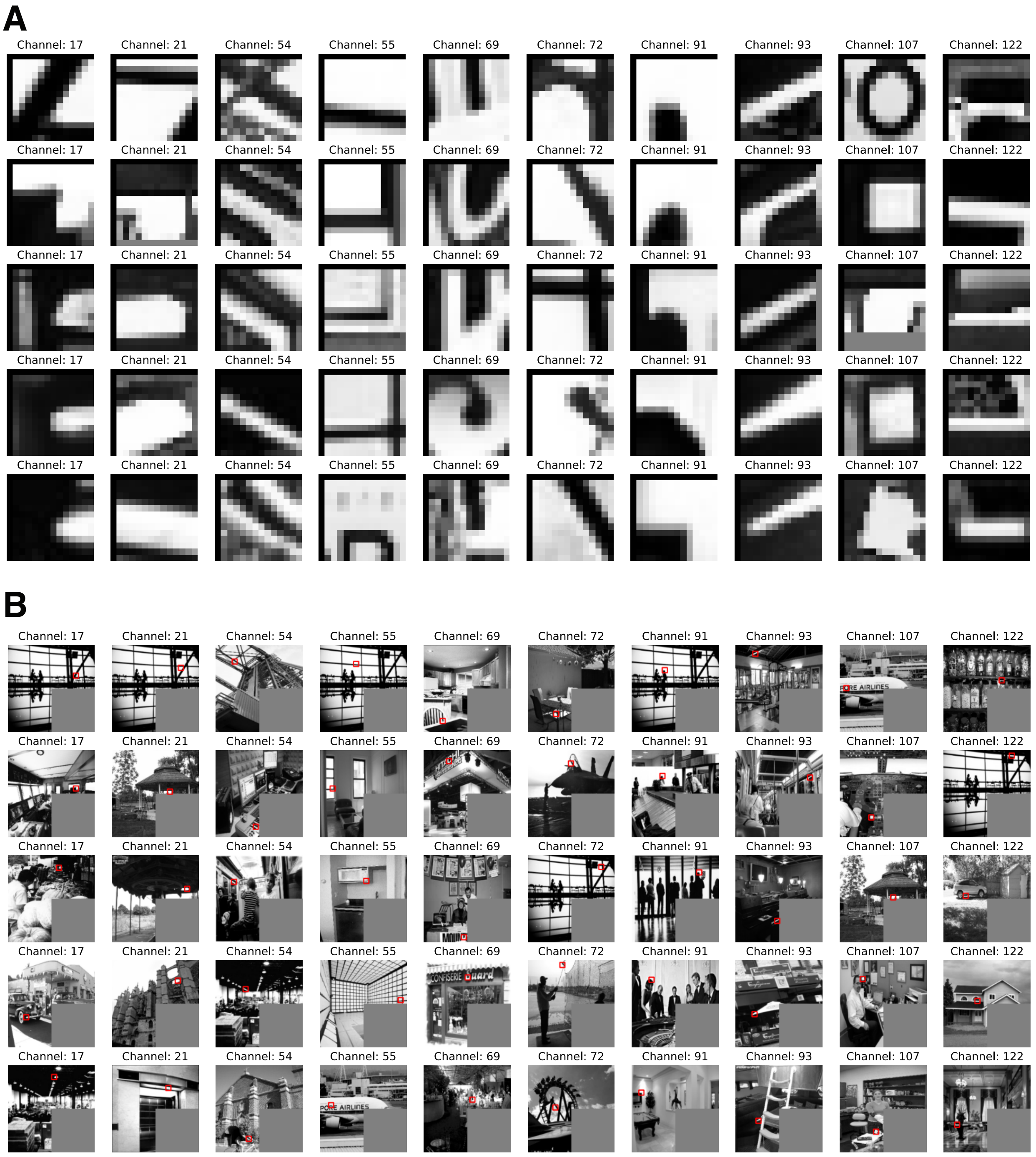

Figure 17: Layer `block2_conv2` max activations. Every column is a channel. **(A)** Receptive fields, in the image space, that provide the five largest activations for a specific channel. **(B)** Original images are provided.

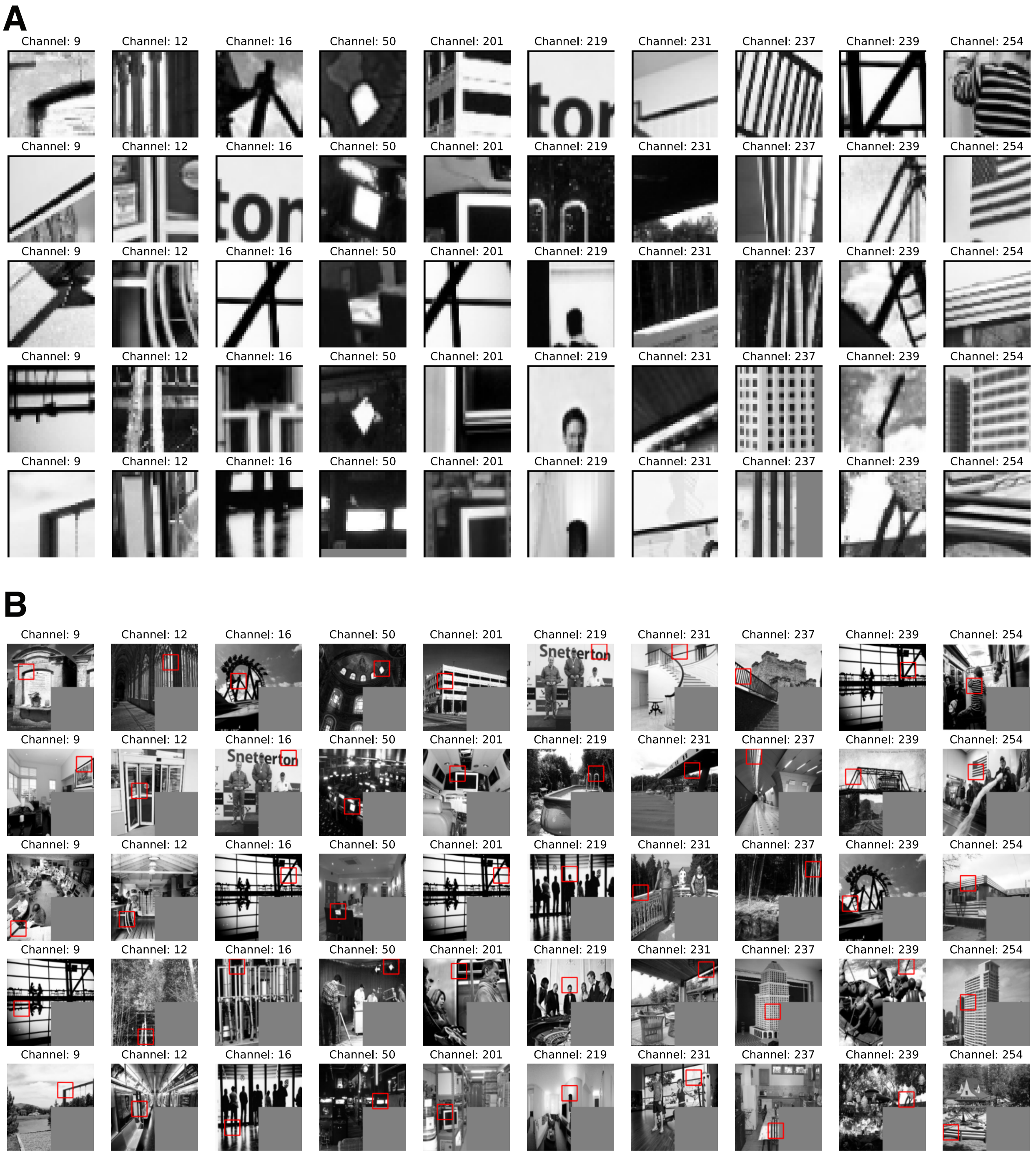

Figure 18: Layer `block3_conv3` max activations. Every column is a channel. **(A)** Receptive fields, in the image space, that provide the five largest activations for a specific channel. **(B)** Original images are provided.

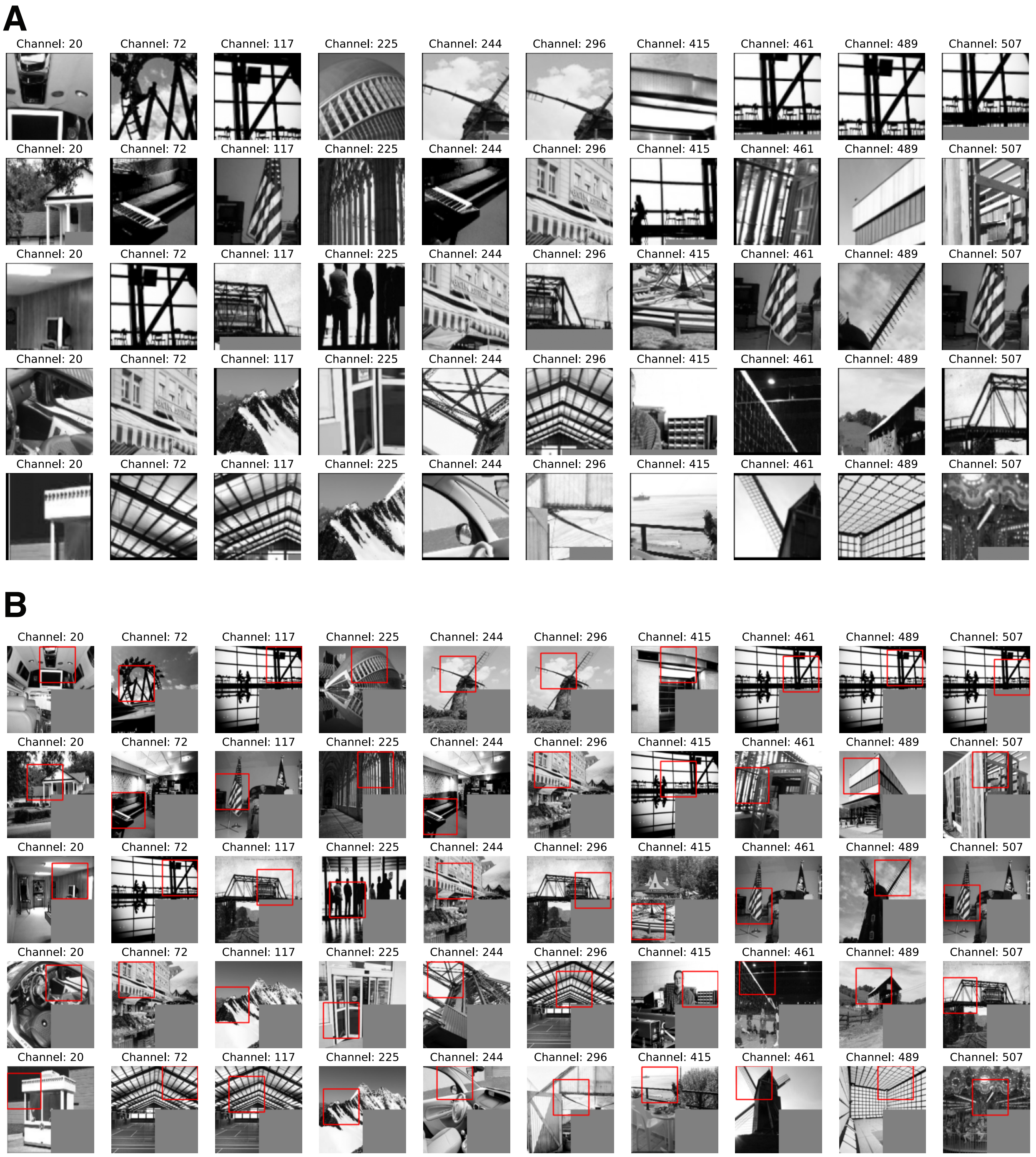

Figure 19: Layer `block4_conv3` max activations. Every column is a channel. **(A)** Receptive fields, in the image space, that provide the five largest activations for a specific channel. **(B)** Original images are provided.

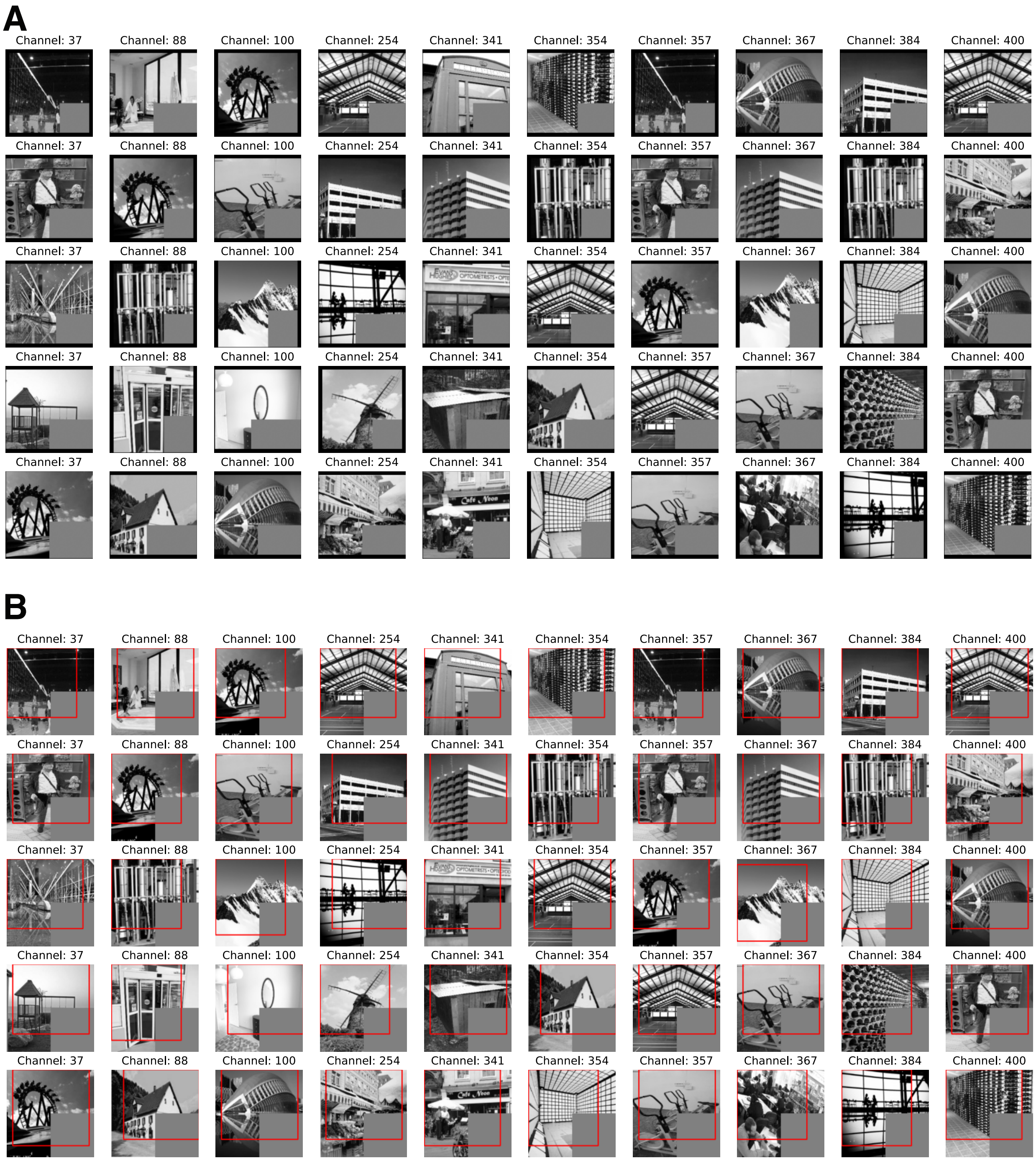

Figure 20: Layer `block5_conv3` max activations. Every column is a channel. **(A)** Receptive fields, in the image space, that provide the five largest activations for a specific channel. **(B)** Original images are provided.
